# Non-coding RNAs associated with Prader–Willi syndrome regulate transcription of neurodevelopmental genes in human induced pluripotent stem cells

**DOI:** 10.1093/hmg/ddac228

**Published:** 2022-09-09

**Authors:** Monika Sledziowska, Kinga Winczura, Matt Jones, Ruba Almaghrabi, Hannah Mischo, Daniel Hebenstreit, Paloma Garcia, Pawel Grzechnik

**Affiliations:** School of Biosciences, University of Birmingham, Edgbaston, Birmingham B15 2TT, UK; School of Biological Sciences, University of Manchester, Michael Smith Building, Oxford Road, Manchester M13 9PT, UK; School of Life Sciences, Gibbet Hill Campus, University of Warwick, Coventry CV4 7AL, UK; Institute for Cancer and Genomic Sciences, University of Birmingham, Edgbaston, Birmingham B15 2TT, UK; School of Immunology & Microbial Sciences, King’s College London, London SE1 9RT, UK; School of Life Sciences, Gibbet Hill Campus, University of Warwick, Coventry CV4 7AL, UK; Institute for Cancer and Genomic Sciences, University of Birmingham, Edgbaston, Birmingham B15 2TT, UK; Birmingham Centre for Genome Biology, University of Birmingham, Edgbaston, Birmingham B15 2TT, UK; School of Biological Sciences, University of Manchester, Michael Smith Building, Oxford Road, Manchester M13 9PT, UK

## Abstract

Mutations and aberrant gene expression during cellular differentiation lead to neurodevelopmental disorders, such as Prader–Willi syndrome (PWS), which results from the deletion of an imprinted locus on paternally inherited chromosome 15. We analyzed chromatin-associated RNA in human induced pluripotent cells (iPSCs) upon depletion of hybrid small nucleolar long non-coding RNAs (sno-lncRNAs) and 5’ snoRNA capped and polyadenylated long non-coding RNAs (SPA-lncRNAs) transcribed from the locus deleted in PWS. We found that rapid ablation of these lncRNAs affects transcription of specific gene classes. Downregulated genes contribute to neurodevelopment and neuronal maintenance, while upregulated genes are predominantly involved in the negative regulation of cellular metabolism and apoptotic processes. Our data reveal the importance of SPA-lncRNAs and sno-lncRNAs in controlling gene expression in iPSCs and provide a platform for synthetic experimental approaches in PWS studies. We conclude that ncRNAs transcribed from the PWS locus are critical regulators of a transcriptional signature, which is important for neuronal differentiation and development.

## Introduction

Prader–Willi syndrome (PWS) is a genetic neurodevelopmental disorder characterized by hypotonia in infancy, developmental delay, cognitive disability, behavioural problems and hyperphagia often leading to life-threatening obesity (1). The cause of PWS is the lack of expression of genes from the paternally inherited locus q11-q13 on chromosome 15. This can occur as a result of a paternal deletion in the 15q11-q13 region (70% of cases), maternal uniparental disomy (20–30% of cases), imprinting defects (1% of cases) as well as rare translocations or microdeletions in the locus (2–5). *Post-mortem* analysis of hypothalamic tissue of patients with PWS revealed excessive expression of genes signalling hunger and microglial genes associated with inflammatory responses as well as downregulation of genes which regulate feeding, neurogenesis, neurotransmitter release and synaptic plasticity (6). The molecular processes resulting in this misregulation of gene expression are yet to be determined.

**Figure 1:**
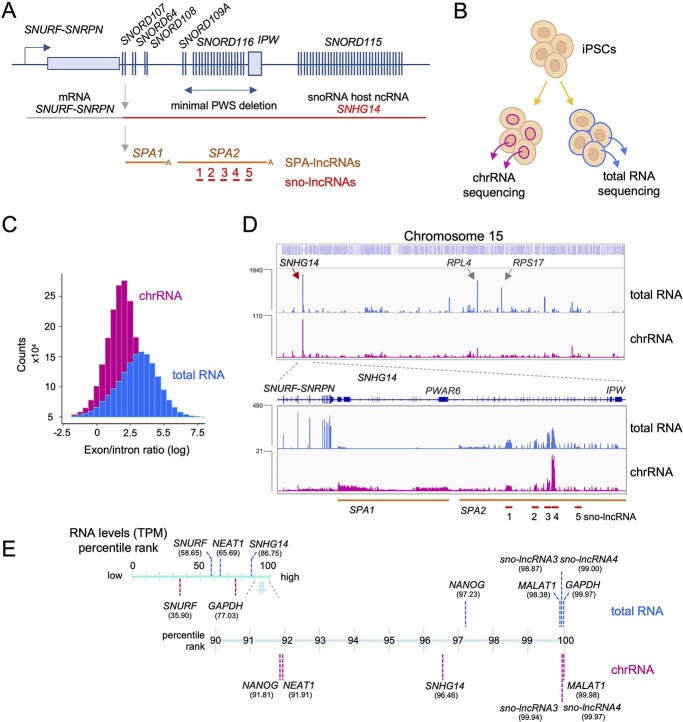
Accumulation of PWS-related ncRNAs on the chromatin in iPSCs. (**A**) Diagram showing organization of lncRNAs transcribed downstream of *SNURF-SNRPN* gene. *SNORD,* snoRNA genes, *IPW,* imprinted gene in the Prader–Willi syndrome region. (**B**) The experimental approach used in the study. RNA was isolated either from the whole cell (total RNA) or from the insoluble nuclear fraction (chromatin-associated RNA). (**C**) Distribution of the size-normalized ratio of RNA-seq reads that map to exons versus introns in total and chromatin-associated RNA fractions. (**D**) Distribution of total RNA (blue) and chromatin-associated RNA (purple) on chromosome 15. Locations of SPA-lncRNAs and sno-lncRNAs are shown below the track; chrRNA-seq analysis. (**E**) The abundance of RNAs in iPSCs in total and chromatin-associated RNA fractions are shown for selected genes as a percentile rank. TPM, transcript per million.

The PWS locus encodes the *SNURF-SNRPN*, *NDN*, *MKRN3*, *NAPAP1* and *MAGEL2* genes. *SNURF-SNRPN* 3′ untranslated region extends into the non-coding gene called *SNHG14* (Fig. 1A). The introns of *SNHG14* contain multiple clusters of box C/D small nucleolar RNAs (snoRNAs) and overlap with several long non-coding RNAs (lncRNAs) called *PWAR* (Prader–Willi/Angelman Region RNA) and *IPW* (Imprinted in Prader–Willi) (1). Both *SNURF-SNRPN* and *SNHG14* share the same promoter. The minimal deletions associated with PWS span either 71 kb or 118 kb in *SNHG14*, and encompass 29 copies of snoRNA *SNORD116*, the *IPW* gene and, in the case of the larger deletion, additionally a single snoRNA, *SNORD109A* (3,4). It is therefore formally possible that the disrupted expression of any of these genes, including full-length lncRNA *SNHG14*, is a direct cause of PWS. Due to their associated enzymatic activity, snoRNAs are the main candidates. Box C/D snoRNAs are short (60–300 nucleotides) ncRNAs that form ribonucleoprotein complexes and mediate ribose 2′-O-methylation of predominantly ribosomal RNA (rRNA) and small nuclear RNAs (snRNAs) (7). These snoRNAs contain guiding sequences which are complementary to their RNA targets. However, the majority of snoRNAs in humans do not possess a clear complementarity to any cellular RNA sequences and thus are called “orphan” snoRNAs. These snoRNAs may act on multiple RNA targets or play other undetermined roles in the cell. Importantly, snoRNAs encoded from the 15q11-q13 locus are also orphan snoRNAs, and their targets and functions remain consequently largely unknown (7).

Recent studies showed that selected snoRNAs from the locus missing in PWS can form two types of hybrid lncRNAs: five small nucleolar RNA related lncRNAs (sno-lncRNAs) and two 5′ snoRNA capped and polyadenylated lncRNAs (SPA-lncRNAs) (Fig. 1A) (8,9). Both sno-lncRNAs and SPA-lncRNAs are by-products of *SNURF-SNRPN-SNHG14* processing. Sno-lncRNA is formed by two snoRNAs embedded into the same intron which, when spliced out, is degraded by 3′-5′ and 5′-3′ exonucleases. Intron degradation continues until it is blocked by the snoRNAs that define the 3′ and 5′ ends of sno-lncRNA. Thus, each of the five sno-lncRNAs consists of an intervening sequence flanked by two snoRNAs at each end, all arising from the same intron of the *SNHG14* ncRNA (8). The 5′ ends of the two SPA-lncRNAs in the PWS locus coincide with *SNORD107* (*SPA1-lncRNA*) and *SNORD109A* (*SPA2-lncRNA*), while the 3′ ends are polyadenylated. SPA-lncRNA formation is associated with the degradation of RNA downstream of the poly(A) site (PAS). Following endonucleolytic cleavage, the RNA is degraded by the 5′-3′ exonuclease until it reaches a snoRNA sequence. This generates the 5′ end of SPA-lncRNA and allows RNA Polymerase II to continue elongation to another PAS sequence that defines the 3′ end of SPA-lncRNA (9). The intervening regions of sno- and SPA-lncRNAs were shown to sequester the RNA processing and splicing factors TDP43, RBFOX2 and hnRNP M and hence affect alternative splicing (8,9).

Previous studies of PWS in cellular models focused primarily on changes in total RNA (6,8–10), which reflects the levels of cytoplasmic steady-state RNA. We examined the possibility that the ablation of sno-lncRNAs and SPA-lncRNAs from the 15q11-q13 locus affects the nascent transcriptome. Since this is usually not detectable in total RNA we examined chromatin-associated RNA (chrRNA) that reflects active nascent transcription across the genome. Acute depletion of sno- and SPA-lncRNAs in human induced pluripotent stem cells (iPSCs) revealed their role in the regulation of transcription of neuronal genes. These observations provide an insight into a potential molecular mechanism by which deletion in the 15q11-q13 region affects neuronal differentiation and thus gives rise to the cognitive and behavioural symptoms occurring in PWS.

## Results

### SPA-lncRNAs and sno-lncRNAs accumulate at high levels in iPSCs

SPA- and sno-lncRNAs were initially described in human embryonic H9 and teratocarcinoma PA1 cell lines (8,9). Thus, to investigate the impact of PWS-related ncRNAs on gene expression in undifferentiated cells, we chose human iPSC CREM003i-BU3C2 line reprogrammed from a blood sample of a 40-year-old male (11). First, we employed reverse transcription-quantitative PCR (RT-qPCR) analysis to determine if SPA- and sno-lncRNAs were expressed in the iPSCs as well as HEK293T and HeLa cell lines derived from human embryonic kidney and cervical cancer cells, respectively, as a negative control. We confirmed that SPA- and sno-lncRNAs were abundantly expressed in iPSCs, while they were barely detectable in either HEK293T or HeLa cells (Supplementary Material, Fig. S1A) as previously reported (8,9). This confirmed our iPSC line as a model suitable for PWS-related research.

SPA- and sno-lncRNAs are localized close to their transcription sites (8,9). Thus, we tested if these ncRNAs were bound to the chromatin in iPSCs by performing chrRNA sequencing (chrRNA-seq) (12). Nuclei were extracted and the soluble nuclear fraction separated from the insoluble chromatin pellet, which retains tightly associated transcription factors and chrRNA, including newly synthesized and nascent RNA (Fig. 1B). In parallel, from intact cells, we isolated total RNA, which is dominated by steady-state, cytoplasmic RNA. Both fractions were prepared in biological duplicates and sequenced on the Illumina platform. The chrRNA fraction showed a clear increase in intronic to exonic reads ratio (median reads ratio exon/intron for chrRNA was 5.75 while for total RNA 32.38), indicative of the efficient removal of steady-state RNA (Fig. 1C and Supplementary Material, Fig. S1B), as previously described (12–14). In the total RNA fraction, RNAs from *SNHG14* locus, encompassing the SPA- and sno-lncRNAs, were one of the most abundant transcripts on chromosome 15, followed by two mRNAs encoding ribosomal proteins *RPL4* and *RPS17* (Fig. 1D). The chromatin fraction showed that transcripts from *SNHG14* were the dominant RNAs from chromosome 15 (Fig. 1D). In particular, *sno-lncRNA3* and *sno-lncRNA4* were most abundant in both total RNA and chrRNA. This was in contrast to data from H9 cells where SPA-lncRNAs were one of the most highly expressed ncRNAs from the PWS locus only second to *SNURF-SNRPN* mRNA (9).

This observation prompted us to test the overall cellular abundance of PWS ncRNAs in iPSCs. We calculated transcript per million (TPM) values for total RNA and chrRNA samples and ranked transcripts by their expression level (Fig. 1E). In total RNA, containing mostly cytoplasmic RNAs, *SNHG14* was within 14% of the top expressed genes (ranked on the 86th percentile), higher than the ubiquitously transcribed lncRNA *NEAT1* (65th percentile). *SNURF-SNRPN* mRNA was ranked in the 58th percentile. One of the most abundant RNAs in the total RNA fraction were *MALAT1* and *GAPDH* (ranked on the 98th and 99th percentile, respectively), confirming the accuracy of our analysis. In the chromatin-associated fraction, *SNHG14* transcript ascended to the top 4% (96th percentile) most abundant RNAs and was ranked higher than pluripotency factor *NANOG* and *NEAT1* (both ranked on the 91st percentile) (Fig. 1E). Similar to the total RNA fraction, *SNURF-SNRPN* chromatin-associated mRNA was ranked much lower than *SNHG14* ncRNA. *Sno-lncRNA3* and *sno-lncRNA4* were ranked in top 2% and 1% of expressed transcripts in total RNA and chrRNA, respectively. Highly expressed genes often carry out critical roles in cells whereas maintaining high levels of non-functional RNAs may have deleterious effects (15); therefore, very high expression of *SNHG14*-derived RNAs may indicate that they play important roles in iPSCs. Discrepancies in mRNA levels between the two fractions, for example for *GAPDH* (99^th^ and 65^th^ in total and chrRNA, respectively), reflect the fact that mRNA levels in the cell are maintained not only by RNA synthesis but also by RNA stability (16).

**Figure 2:**
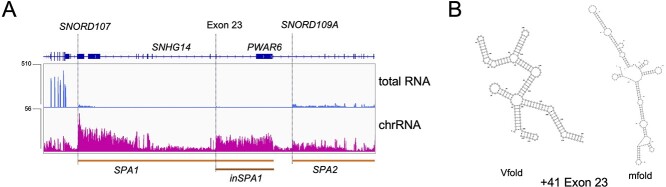
Putative novel lncRNA in the PWS region. (**A**) Distribution of chrRNA-seq reads indicates the position of the putative additional lncRNA (*inSPA1*). (**B**) Secondary RNA structure of exon 23 revealed by Vfold and mfold predictions. chrRNA-seq tracks show counts ×10^6^; chrRNA, chromatin-associated RNA.

Our analysis revealed that almost all PWS ncRNAs were clearly detected in both the total RNA and chrRNA fractions, with the exception of *SPA1-lncRNA*, which was less abundant in the total RNA than other transcripts (Fig. 1D). Moreover, we detected increased reads spanning from position +41 of exon 23 to the *PWAR6* in *SNHG14*, within the boundaries of the *SPA1-lncRNA* (Fig. 2A). This suggests the presence of a previously unannotated ncRNA, which we term ‘inside-of-SPA1-lncRNA’ (*inSPA1*), that may arise from 5′-3′ degradation of *SPA1-lncRNA* if exonucleolytic degradation is blocked by RNA structures further downstream. Computational prediction using Vfold and mfold software (17,18) revealed that the 5′ end of *inSPA1-lncRNA*, containing exon 23 and its 41 upstream nucleotides, indeed may fold into several stem loops that may be able to block exonucleolytic trimming (Fig. 2B).

### Antisense oligonucleotides-dependent depletion of PWS transcripts

**Figure 3:**
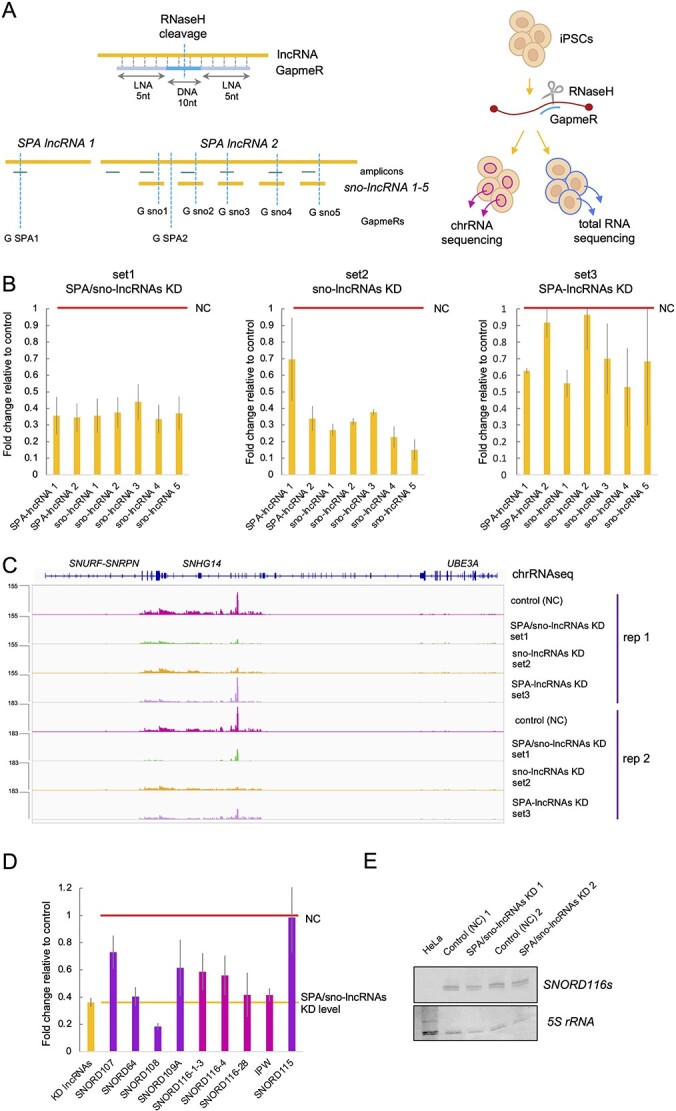
Antisense oligonucleotides mediate efficient knockdown of SPA- and sno-lncRNAs. (**A**) The locations of GapmeRs (vertical blue lines denoted G) targeting SPA- and sno-lncRNAs and qPCR amplicons (horizontal green lines) used in the study. Note the overlap of sno-lncRNAs and *SPA2-lncRNA*. A diagram depicting the experimental design is shown on the right. (**B**) Fold change relative to negative control (red line) in SPA- and sno-lncRNAs levels 24 h post introduction of GapmeRs into iPSCs. RT-qPCR analysis showing an average of three independent experiments; error bars correspond to standard error. (**C**) chrRNA-seq reads from SPA/sno-lncRNAs host gene *SNHG14* in iPSCs treated with different sets of GapmeRs. Rep, replicate. chrRNA-seq tracks show counts ×10^6^. (**D**) Fold change relative to negative control (red line) in levels of snoRNAs and *IPW* processed from *SNHG14*. RNA was isolated 24 h post introduction of GapmeRs into iPSCs. RT-qPCR analysis showing an average of four independent experiments (including samples used for RNA-seq); error bars correspond to standard error. (**E**) Mature *SNORD116s* visualized by northern blot. Two dominant 92 and 95 nt long forms of *SNORD116* were detected. *5S* rRNA visualized by methylene blue staining is shown as a loading control. RNA isolated from HeLa cells was used as negative control. Uncropped images are shown in Supplementary Materials.

Fast depletion approaches provide an opportunity to investigate the most direct consequences of how the reduced ncRNA levels affect cellular pathways. To determine the effect of an acute ablation of SPA- and sno-lncRNAs on the transcriptome, we employed antisense oligonucleotides (ASO) GapmeRs (Qiagen), which targeted lncRNAs and triggered RNA cleavage by endogenous RNase H and the subsequent degradation by exoribonucleases. We designed a panel of GapmeRs against the individual PWS ncRNAs (Fig. 3A) targeting their intervening sequences located in-between terminal snoRNAs or poly(A) tail. To compare the effects of different types of ncRNAs on transcription, we used three sets of GapmeRs against: (1) all seven sno/SPA-lncRNAs, (2) five sno-lncRNAs and (3) two SPA-lncRNAs. These were compared with negative control, where iPSCs were treated with an equivalent amount of non-specific GapmeRs. The GapmeR against *SPA2-lncRNA* was designed not to affect any sequences contained within sno-lncRNAs. However, all GapmeRs against sno-lncRNAs also targeted *SPA2-lncRNA*.

We nucleofected iPSCs with each set of GapmeRs and isolated total RNA and chrRNA fractions after 24 h. RT-qPCR performed on total RNA revealed efficient knockdown of all seven lncRNA species using GapmeRs set 1 (SPA/sno-lncRNAs KD) (Fig. 3B). In cells transfected with set 2 (sno-lncRNAs KD), both sno-lncRNAs and *SPA2-lncRNA* were knocked down. Set 3 (SPA-lncRNAs KD) knocked down *SPA1-lncRNA*; however, *SPA2-lncRNA* was only partially affected. Overall, we achieved a knockdown up to 80% (65% on average) of the selected RNA species across the different sets. We also tested the efficiency of GapmeRs by quantifying chromatin-associated *SNHG14* ncRNAs via chrRNA-seq upon GapmeR nucleofection (Fig. 3C). Our data revealed that GapmeRs set 1 efficiently reduced accumulation of *SPA1-lncRNA* (and putative *inSPA1*), *SPA2-lncRNA* and all five sno-lncRNAs. GapmeRs set 2 decreased sno-lncRNAs and *SPA2-* but not *SPA1-lncRNA*, while set 3 mainly affected *SPA1-lncRNA* and to a lesser extent *SPA2-* and sno-lncRNAs. Interestingly, GapmeRs-dependent knockdown was still detectable for some RNAs after 5 days from nucleofection (Supplementary Material, Fig. S2A), demonstrating the utility of this system in longer experimental setups.

To assess the specificity of the GapmerR treatment, we also assessed how other RNA species processed from *SNHG14* were affected by multiple GapmeRs-dependent cleavages. RT-qPCR analysis revealed that the levels of *SNORD107, 64, 108* and *109A* were reduced by 27, 60, 81 and 39%, respectively. *SNORD115s* which are located further downstream of the cleavage sites were unaffected by the GapmeRs (Fig. 3D). Of 29 copies of *SNORD116s*, 10 are included into sno-lncRNAs. To assess the levels of *SNORD116s* not included into any sno-lncRNAs, we took advantage of minor sequence variabilities in *SNORD116* sequences and designed primer pairs fully complementary only to either *SNORD116-1-3*, *SNORD116-4* or *SNORD116-28*. The levels of these *SNORD116s* were reduced by 42, 45 and 59%, respectively, while SPA/sno-lncRNAs were reduced by 65% on average (Fig. 3D). Even though we operated at stringent PCR-annealing temperatures, we cannot fully exclude that the primer pairs also recognized other *SNORD116s*. We therefore employed a northern blot analysis to test overall *SNORD116s* levels using denaturing UREA-PAGE and a probe (95 nt long *SNORD116* sequence) against all *SNORD116s*. This northern blot analysis detected two dominant 92 nt and 95 nt long types of *SNORD116s* which assured us that we investigated a range of *SNORD116s* from the cluster. This analysis revealed that the levels of mature *SNORD116s* were not affected by SPA/sno-lncRNAs depletion (Fig. 3E and Supplementary Material, Fig. S2B). The differences in RT-qPCR and northern blot analysis implicate that most of *SNORD116s* may exist in iPSCs in unprocessed form, ‘trapped’ in *SNGH14* ncRNAs. The level of non-coding *IPW* was reduced similarly to SPA/sno-lncRNAs (Fig. 3D). However, about 40% of *IPW* is included in *SNHG14* (Supplementary Material, Fig. S2C) and therefore the decrease may reflect increased degradation of *SNHG14* in the upstream region as observed for snoRNAs.

### SPA- and sno-lncRNAs regulate transcription of neuronal genes

Differential expression analysis did not detect global effects on the steady-state transcriptome in iPSCs nucleofected with GapmeRs set 1 targeting all PWS lncRNAs (Supplementary Material, Fig. S3A). This is consistent with earlier reports showing only small changes in alternative splicing in the stem cells lacking SPA- and sno-lncRNAs (8,9) but contrary to other studies of *post-mortem* hypothalamic tissues of PWS patients revealing that genes regulating neuronal processes and genes contributing to immune response were affected (6). Since many ncRNAs control transcription of protein-coding genes (19), we performed the sequencing of chrRNA, as a proxy of active transcription, upon SPA- and sno-lncRNAs knockdowns to understand the processes driving PWS. ChrRNA-seq has been used extensively to investigate transcriptional processes and proved as a reliable approach in the assessment of the transcription of protein-coding genes (12–14,20–22).

**Figure 4:**
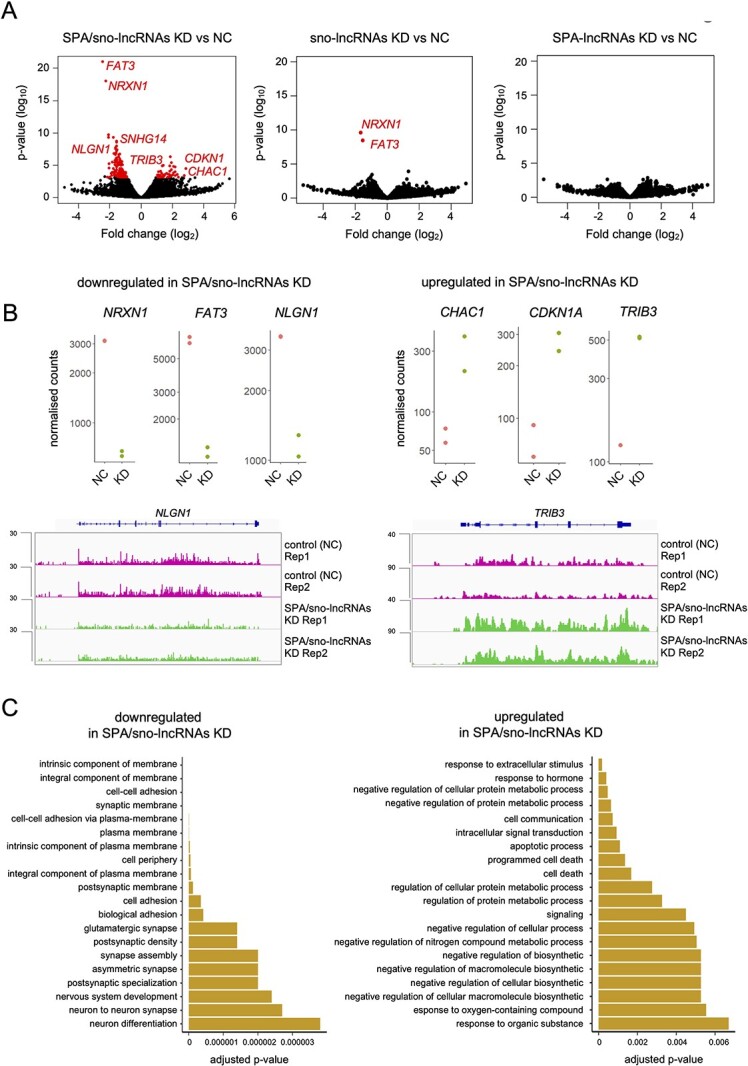
Depletion of SPA- and sno-lncRNAs affects the accumulation of chromatin-associated RNA in iPSCs. (**A**) Differentially accumulated chromatin-associated RNAs (in red) between the control (NC, nonspecific GapmeRs control) and SPA/sno-lncRNAs knockdown (KD) combinations. Volcano plots showing results of two biological replicates of chrRNA-seq for each sample. (**B**) Normalized counts for genes selected from the top 10 either down- or up-regulated genes in chrRNA-seq analysis of SPA/sno-lncRNAs depletion. chrRNA-seq reads coverage for *NLGN1* and *TRIB3* in SPA/sno-lncRNAs KD and control are shown below. chrRNA-seq track shows counts ×10^6^. (**C**) GO terms associated with downregulated and upregulated genes in chrRNA-seq datasets upon depletion of SPA/sno-lncRNAs. chrRNA, chromatin-associated RNA.

Our chrRNA-seq performed on cells treated with GapmeRs sets for 24 h uncovered the impact of PWS ncRNAs on transcription of many protein-coding genes (Fig. 4A). We used differential gene expression analysis to compare SPA/sno-lncRNAs KD (set 1), sno-lncRNAs KD (set 2) and SPA-lncRNAs KD (set 3) with a control treated with non-specific GapmeRs, all in two biological repeats. We observed the greatest alterations in RNA levels between SPA/sno-lncRNAs KD and control conditions, with 205 downregulated and 87 upregulated transcripts. The differences observed between the remaining two conditions and control were more subtle, which is consistent with the varied efficiency of the GapmeR sets in depletion of PWS ncRNAs. Among the top 10 most downregulated genes in SPA/sno-lncRNAs KD were *FAT3*, *NRXN1* and *NLGN1* (Fig. 4A and B and Supplementary Material, Fig. S3B). These downregulated genes are involved in the regulation of neuronal development and function; FAT3 is a cadherin that determines the polarity of developing neurons by regulating the interactions between neurites (23), while NLGN1 and NRXN1 are membrane adhesion proteins, which have the capacity to bind to each other across a synapse. NLGN1 induces the formation of presynaptic boutons allowing for neuron maturation (24), and NRXN1 regulates neuronal development, function and synaptic transmission (25,26). The downregulation of most affected genes identified in chrRNA-seq was validated by the RT-qPCR analysis of chrRNA (Supplementary Material, Fig. S3D). The comparison between sno-lncRNAs KD (set 2) and control rendered only two downregulated genes, *FAT3* and *NRXN1*, while the SPA-lncRNAs KD (set 3) failed to produce any significant changes in transcription (Fig. 3A). Interestingly, *NRXN1* was the only mRNA which increased in the total RNA fraction upon depletion of PWS ncRNAs. We speculate that this may be a result of a compensation effect (27–29), a response to the overall decrease in transcription of other genes involved in neurodevelopment; however, more research is needed to explain this observation. Noteworthy, both increased and decreased expression of *NRXN1* is associated with neurological diseases (30,31).

Among the most upregulated genes by SPA/sno-lncRNAs KD in the chromatin-associated fraction were genes that negatively affect proliferation and differentiation, or contribute to neuronal function: *CDKN1A*, *JDP2*, *CHAC1* and *TRIB3* (Fig. 4B and Supplementary Material, Fig. S3C). CDKN1A negatively affects cellular proliferation by binding to and inhibiting cyclin-dependent kinases activities (32,33). JDP2 is involved in transcriptional responses associated with transcription factor AP-1, such as induced apoptosis and cell differentiation (34). CHAC1 inhibits Notch signalling promoting neuronal differentiation, while TRIB3 is an inactive kinase that plays a role in programmed neuronal cell death (35,36). Two other knockdowns of sno-lncRNAs and SPA-lncRNAs (set 2 and 3) did not result in transcriptional upregulation for any genes (Fig. 4A). Consistent with the results of differential expression analysis, hierarchical clustering correctly grouped the duplicates of the SPA/sno-lncRNAs KD, sno-lncRNAs KD and control samples. The SPA-lncRNAs KD sample duplicates did not cluster well, which may be the result of less efficient and variable knockdowns (Supplementary Material, Fig. S4A).

Active transcription of genes involved in neuronal differentiation in iPSCs was somewhat unexpected. Thus, we sought to confirm the stem status of the CREM003i-BU3C2 cell line and tested for the presence of actively transcribed neuronal markers. We calculated the abundance of transcripts in chrRNA isolated from cells nucleofected with control GapmeRs. Transcription of proliferation markers including *POU5F1*, *NANOG* and *SFRP2* was evident in the chrRNA-seq (Supplementary Material, Fig. S4B and Table S1). Markers of both immature neurons (e.g. *NEUROD1, NCAM1, DCX*) and mature neurons (e.g. *ENO2, MAP2, TUBB3, NEFL*) were detected to a lesser extent in the chromatin-associated RNA fraction, and markers for functional neurons (e.g. *CHAT, TH, GAD2*) were virtually absent. A similar trend was observed in the total RNA fraction (Supplementary Material, Fig. S4C). The accumulation of mRNA of genes involved in neurodevelopment *NRXN1*, *NLGN1* and *FAT3* in total RNA fraction was significantly lower than in the chromatin-associated RNA, which was the opposite pattern observed for constitutively expressed gene *GAPDH* (Supplementary Material, Fig. S4D). This indicates that these were transcribed but their mRNAs did not accumulate in non-differentiating iPSCs.

Next, we focused on exploring the functions of differentially expressed genes upon combined SPA/sno-lncRNAs depletion. We employed Gene Ontology (GO) terms analysis to identify the relevant pathways. The genes whose transcription was downregulated by the SPA/sno-lncRNAs knockdown are involved in pathways related to neuronal development, function and cell adhesion (Fig. 4C), which is consistent with the decreased levels of *FAT1*, *NRXN1* and *NLGN1* in this condition (Fig. 4B). These pathways include maintenance of membrane integrity, glutamatergic synapses, synaptic membranes and synapse assembly processes (all *P*-values < 0.001). Genes that were upregulated by the SPA/sno-lncRNAs knockdown participate in the dampening of cellular growth and proliferation as well as in promoting apoptosis, including regulation of cellular protein metabolic processes, apoptotic processes and programmed cell death (*P*-value < 0.001) (Fig. 4C). This is consistent with transcriptional upregulation of *CDKN1A*, *TRIB3* and *JDP2* in this knockdown (Fig. 4B).

**Figure 5:**
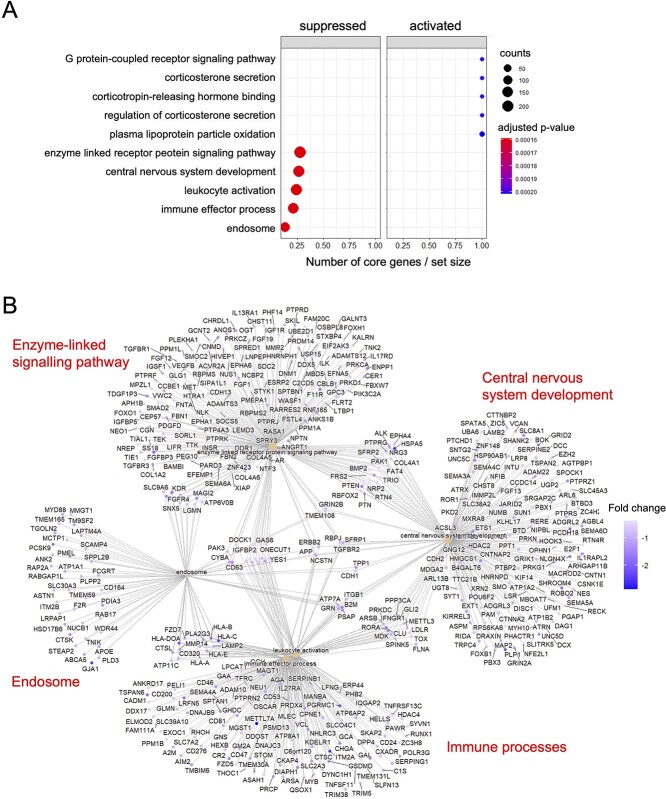
SPA- and sno-lncRNAs impact multiple pathways in iPSCs. (**A**) Activated and suppressed pathways indicated by changes in chromatin-associated RNA fractions upon SPA/sno-lncRNAs knockdown. (**B**) Cnetplot showing genes participating in the top five supressed pathways by SPA- and sno-lncRNAs knockdown.

To complement the GO analysis, we employed Gene Set Enrichment Analysis. The input for this analysis consisted of all detected transcripts, which allowed for identifying small, coordinated changes that would not otherwise be recognized (37). These transcripts were then ranked based on the fold change which allowed for the determination of a wide range of activated or suppressed pathways (Fig. 5A). The results of this analysis were consistent with the GO results, revealing pathways associated with the central nervous system development (*P*-value < 0.001) were among those suppressed upon SPA/sno-lncRNAs knockdown. This method allowed us to identify a number of other affected pathways, which were not evident from the GO analysis. We found that SPA/sno-lncRNAs depletion also suppresses pathways associated with intracellular and cell–cell signalling (correlated with terms ‘enzyme linked receptor protein signalling pathway’ and ‘endosome’), as well as processes linked to immune processes (terms ‘leukocyte activation’, ‘immune effector process’), which is consistent with the misregulation of immune system genes observed in PWS patients (6). We further identified a group of activated pathways that are involved in hormonal regulation. However, these pathways included fewer genes and were associated with higher *P*-values than suppressed pathways. Genes identified in the top five most significant pathways were visualized as cnetplot (Fig. 5B). Interestingly, the genes contributing to the suppressed pathways that regulate the development of the central nervous system included *SHANK2* and *CNTNAP2*, deletions of which are associated with autism and intellectual disability (38,39). Overall, our analyses indicate that the lack of ncRNAs transcribed from the locus deleted in PWS may deregulate a broad spectrum of genes involved in pathways that may contribute to aberrant development of the structures in the human brain.

## Discussion

The molecular basis of the neurodevelopmental genetic disorder PWS remains largely unknown. We depleted SPA-lncRNAs and sno-lncRNAs transcribed from the 15q11-q13 locus deleted in PWS to study their impact on transcription in human iPSCs. Our analysis of chrRNA shows that the lack of these ncRNAs decreased transcription of neurodevelopmental genes and increased transcription of factors that negatively affect cellular growth and mediate apoptosis. The region downstream of the *SNURF-SNRPN* gene, *SNHG14*, that encompasses the minimal deletion resulting in PWS (3–5) produces one of the most abundant chrRNAs in iPSCs (Fig. 1). This is consistent with previous reports showing that SPA- and sno-lncRNAs levels are among the most highly expressed lncRNAs in embryonic H9 cells (8,9). The chromatin association of PWS ncRNAs somewhat points towards their potential function in transcription regulation; approximately 60% of lncRNAs display a strong bias for the chromatin association and play roles in the activation of neighbouring or distant genes (19). SPA- and sno-lncRNAs display unique structural properties: the snoRNA structures at the 5′ (in case of SPA-lncRNAs) or both (in case of sno-lncRNAs) ends make them relatively resistant to exonucleolytic degradation. Thus, the intervening sequence can be used to sequester transcription and splicing factors TDP43, RBFOX2 and hnRNP M and hence affect regulation of alternative splicing (8,9). Moreover, RBFOX2 can also impact transcription by recruiting to the nascent RNA the silencing polycomb group complex PRC2 that represses transcription via trimethylation of histone H3 at lysine 27 (H3K27me3) (40).

Our analysis of chrRNA, which can be used as a proxy for active transcription of protein-coding genes (12), uncovered that SPA- and sno-lncRNAs control a set of genes that regulate neurodevelopment. We found that SPA- and sno-lncRNAs depletion decreased transcription levels for genes that contribute to the formation of neuron specific structures—axons and synapses, as well as genes required for proper cell adhesion and cell–cell signalling (Figs 4 and 5). The presence of genes involved in neuron maturation and maintenance such as *NRXN1*, *NLGN1* or *FAT3* in the chromatin-associated fraction indicates their active transcription (Fig. 3A and B) which was not reflected at similar levels in the steady-state RNA (Supplementary Material, Fig. S4D). The residual transcription may be associated with the bivalent chromatin state (41) which possesses both activating H3K4me3 and repressive H3K27me3 marks, a characteristic of stem cells (42). Such a bivalent chromatin environment maintains the gene expression at minimal level allowing for rapid activation in response to stimuli received during the development (43,44). Our chrRNA-seq indicates that SPA- and sno-lncRNAs may be required to maintain the correct transcription of neurodevelopmental genes. Such regulation may be directly provided by the factors that bind to SPA- and sno-lncRNAs and affect chromatin, like RBFOX2 (40). Interestingly, a significant population of genes, which were upregulated by SPA- and sno-lncRNAs knockdown, are involved in apoptosis and negative regulation of cellular metabolism. One potential explanation of this phenotype is that misregulation of neurodevelopmental genes that may result in abnormal differentiation is countered by repression of cell growth and, ultimately, cell death. Indeed, neurons developed from iPSCs that model neurodevelopmental diseases including Friedreich’s ataxia and spinal muscular atrophy are more prone to senescence and apoptosis (45,46).

Noteworthy, *sno-lncRNA5* contains a 298 nt long SINE (short interspersed nuclear element) sequence *AluSx1* positioned in a reverse direction (Supplementary Material, Fig. S5). Similar *Alu* sequences are also present in the introns of some up- and downregulated genes detected in our analysis. SINE elements located in lncRNAs may have functional purposes, e.g. they may facilitate RNA–RNA or RNA–DNA interaction with complementary SINE elements or contribute to lncRNAs nuclear retention (47–49). Thus, more research is needed to understand if *sno-lncRNA5* SINE located in otherwise transposon-depleted *SNORD116* cluster plays any regulatory roles.

The observed changes in transcription may be also caused by the disruption of other RNAs from the *SNHG14* locus, in particular *SNORD116s* which are included into the shortest deletion associated with PWS (3,4). Our northern blot analysis showed that the level of mature *SNORD116s* was not changed by the SPA/sno-lncRNAs depletion indicating that the transcriptional downregulation of neurodevelopmental genes was rather caused by the lack of other PWS ncRNAs. Interestingly, RT-qPCR analyses revealed a decrease of *SNORD116s* level, opposite to results obtained by northern blot. However, in RT-qPCR analysis we tested the levels of both unprocessed (included into introns) and processed (excised from the introns) *SNORD116s*. LncRNAs like *SNHG14* are not efficiently spliced (50), which is required for snoRNA processing and release from the host RNA (7). Thus, the decrease in *SNORD116* level observed in RT-qPCR analysis implies that most of these snoRNAs are not processed from *SNHG14* in iPSCs and the reduction may reflect overall increased RNA degradation in regions proximal to GapmeRs-dependent cleavages. Consistently, the accumulation of *SNORD115s* (processed and unprocessed) located ~ 60 kb from *SNORD116* cluster was not affected by GapmeRs treatment. The minimal deletions causing PWS also include *IPW* non-coding gene. The GapmeRs mediated knockdown reduced *IPW* levels similarly to SPA/sno-lncRNAs; therefore, we cannot exclude that the effects on transcription were caused by the lack of this ncRNA.

The global effect of SPA- and sno-lncRNAs depletion on RNA levels was completely lost in the total RNA fraction, representing mainly steady-state cytoplasmic RNA (Supplementary Material, Fig. S3A, Fig. S2A). This is consistent with the previous observations in PA1 and H9 cells (8,9) as well as resembles a mouse model where only seven genes including transcription factor *Mafa* and growth suppressor *Necdin* were upregulated in brain tissue by deletions in PWS locus (10). However, PWS sno-lncRNAs seem to be specific for primates and their presence has not been confirmed in mouse yet, although the mouse *Snhg14* gene does contain introns with two or more embedded *Snord116s* (51,52). In human cells, deregulation of transcription caused by the absence of SPA- and sno-lncRNAs may feature in total RNA levels in later stages of neurodevelopment, when the expression of these genes is essential to support neuronal maturation. However, it is not clear how the accumulation of transcripts from PWS locus changes during neurodevelopment. Adjusting mRNA to optimal concentration in dynamically differentiating stem cells, when the gene transcription is affected, may not be as responsive and efficient as in healthy cells. Thus, it may introduce errors in gene expression that accumulate during development and, as a consequence, manifest as PWS. Such a pathological pattern, where a transcriptional regulation imbalance emerges already in stem cells, impacts neuronal development and results in a late onset disease, which has been reported for many neurodevelopmental disorders including Fragile X and Rett syndromes as well as neurodegenerative Alzheimer’s and Huntington’s diseases (53,54). As neurons progress through highly organized processes of differentiation, migration and functional activation, the disruption in mRNA synthesis may affect the development of human brain, leading to the manifestation of PWS.

## Materials and Methods

### Cell culture media

Human iPSC line CREM003i-BU3C2 (11) originating from a blood sample of a 40-year-old male was kept at 36°C and 0.5% CO_2_. The cells were cultured in 6-well plates coated with Matrigel (Corning), with StemFlex medium (Gibco) supplemented with Primocin (InvovGen). The media was changed 48 h after a passage and then every 24 h. Cells were passaged using STEMPRO EZPassage tool (Thermo Fisher Scientific) when 70–80% confluent. HEK293T and HeLa transformed cell lines were derived from human embryonic kidney and cervical cancer cells, respectively. Both were cultured at 36°C and 0.5% CO_2_, in 10 cm plates, with DMEM medium (Gibco) supplemented with 10% FBS and penicillin–streptomycin antibiotics mixture (Gibco). Media was changed every 3–4 days.

### GapmeR design

Antisense oligonucleotides (GapmeRs) were designed using QIAGEN online tool with intervening sequences of SPA- and sno-lncRNAs as input. GapmeRs for each ncRNA were selected based on the QIAGEN’s design score, and they were synthesized by QIAGEN. Sequences are listed in Supplementary Material, Table S2.

### Gapmer-mediated knockdown

Confluent iPSCs were treated with 1 ml of TrypLE (Gibco) per well in 6-well plates in order to obtain a single-cell suspension. Cells were incubated for 2 min at 36°C when TrypLE was removed, and then cells were placed back at 36°C for another 3 min. Approximately 1 ml of DPBS (Gibco) was added to each well and the cells were collected and centrifuged at 200 g for 5 min. The supernatant was removed and the cells were resuspended in 100 μl of nucleofector solution from P3 Primary Cell 4D-Nucleofector Kit (Lonza) per transfection. The cells were then divided into individual nucleofection cuvettes, and the appropriate mixture of GapmeRs were added, 6 μl of each. Approximately 2 × 10^6^ cells were utilized per transfection. The transfections were performed using 4D-Nucleofector (Lonza) using setting DS-150. Following the nucleofection the cuvettes were incubated at 36°C for 5 min, then transferred to 1 ml of StemFlex medium and incubated at 36°C for another 10 min. The cells were plated on a Matrigel-coated 6-well plate, and cells from every nucleofection were equally divided between the 6 wells in a plate. The cells were then cultured using standard conditions detailed above for 24 h after which they were collected for RNA extraction.

### RNA extraction and fractionation

The cells were treated with 1 ml of TrypLE (Gibco) per well in a 6-well plates in order to obtain a single-cell suspension. The cells were incubated for 5 min at 36°C or until they detached, at which point 2 ml of DPBS was added per well. The cells were collected and centrifuged at 200 g for 5 min. The supernatant was removed and the cells were either resuspended in 1 ml of TRIZOL (Thermo Fisher Scientific) for total RNA extraction or in 200 μl of Cytoplasmic Lysis Buffer (0.15% NP-40, 10 mM Tris–HCl pH 7, 150 mM NaCl, 50 U RiboLock) for chrRNA fractionation.

For chrRNA fractionation, samples were incubated for 5 min on ice in the Cytoplasmic Lysis Buffer, after which they were layered on 500 μl of Sucrose Buffer (10 mM Tris–HCl pH 7, 150 mM NaCl, 25% Sucrose, 50 U RiboLock). Nuclei were collected by centrifugation of 16 000 g for 10 min at 4°C. The supernatant containing cytoplasmic fraction was then removed, and nuclei were washed with Nuclei Wash Buffer (PBS supplemented with 0.1% Triton X-100, 1 mM EDTA, 50 U RiboLock) at 1200 g for 1 min at 4°C. The supernatant was discarded and the nuclei were resuspended in 200 μl of Glycerol Buffer (20 mM Tris–HCl pH 8, 75 mM NaCl, 0.5 mM EDTA, 50% glycerol, 0.85 mM DTT, 50 U RiboLock). Next 200 μl of Nuclei Lysis Buffer (1% NP-40, 20 mM HEPES pH 7.5, 300 mM NaCl, 1 M Urea, 0.2 mM EDTA, 1 mM DTT, 50 U RiboLock) was mixed with samples by pulsed vortexing for 2 min, and centrifuged at 18 500 g for 2 min at 4°C. The pellet containing chrRNA was resuspended in 200 μl of PBS supplemented with 50 U RiboLock. Following resuspension, 500 μl of TRIZOL was added and the samples were vortexed.

At this point, for both total RNA and chrRNA samples, 100 μl of chloroform was added and the samples were incubated at room temperature for 5 min. The samples were then centrifuged at 16 000 g for 15 min at 4°C. The samples were then processed using RNeasy kit (QIAGEN), following the ‘RNA clean-up’ protocol enclosed with the kit. The samples were then quantified using NanoDrop spectrometer and gDNA contamination was removed using TURBO DNA-free Kit (Thermo Fisher Scientific) according to the manufacturer’s instructions. The samples were stored at −80°C until they were further processed for RT-qPCR or RNA sequencing.

### RT-qPCR

The gDNA-depleted RNA samples were reverse transcribed using SuperScript III Reverse Transcriptase (Thermo Fisher Scientific). Briefly, 500–2000 ng of RNA was diluted up to 11 μl of RNAse-free water, combined with 1 μl of Random Hexamer Primers (Thermo Fisher Scientific) and 1 μl of 10 mM dNTPs and incubated at 65°C for 5 min. Following the incubation, the samples were briefly placed on ice, and 4 μl of SuperScript III Reverse Transcriptase buffer (Thermo Fisher Scientific), 2 μl of DTT, 8 U of RiboLock and 1 μl of SuperScript III Reverse Transcriptase were added per sample. The samples were incubated at 25°C for 10 min, at 50°C for 40 min and at 82°C for 5 min in a thermocycler. For RT-qPCR, the cDNA samples were diluted 1:10. For each 15 μl reaction, 7.5 μl of 2X SyGreen Mix (PCRBio), 0.8 μl of each 10 μM primer, 0.9 μl of PCR grade water and 5 μl of diluted cDNA were mixed. Three reactions per sample and per set of primers were prepared and processed using RotoGene (QIAGEN) with standard cycling. Oligonucleotides used in the study are listed in Supplementary Material, Table S2.

### Northern blot analysis

Approximately 6 μg of total RNA was mixed in 50% formamide, 0.001% Bromophenol blue, incubated for 10 min at 65°C followed by 5 min on ice. The samples were loaded and separated on 6% acrylamide/bis-acrylamide (19:1) gel containing 6 M Urea 1x TBE. RNA was transferred to nylon membrane using semi-dry transfer TransBlot BioRAs (0.5x TBE, 100 mA for 60 min) and crosslinked by UV in crosslinker for 2 min. RNA was stained on the blot using methylene blue solution and destained in 1% SDS. The membrane was pre-hybridized for 30 min in PerfectHyb buffer (Sigma) and incubated overnight with a probe generated by PCR using primers SNORD116 1F and SNORD116 1R and [alpha-P32]GTP. The membrane was washed three times with 2x SSC. Hybridisation of the probe was visualized by autoradiography.

### RNA sequencing

Prior to RNA sequencing, total RNA samples were rRNA depleted using RiboCop (Lexogen) according to the manufacturer’s instructions. Samples were re-quantified using Qubit (Thermo Fisher Scientific), and up to 100 ng of RNA was used for library preparation using NEBNext Ultra II RNA Library Prep Kit for Illumina (NEB). The libraries were prepared according to the manufacturer’s instructions, quantified and quality checked using TapeStation (Agilent). The libraries were prepared with indexing primers and pooled into two library preps. There were eight chrRNA libraries that were pooled together and sequenced on a single G NextSeq 500/550 (150) flow cell to obtain sequencing depth of approximately 40 M reads per sample. The 4 total RNA libraries were pooled with another 8 libraries not analyzed here; the 12 libraries were then loaded and sequenced on another G NextSeq 500/550 (150) flow cell resulting in a lower sequencing depth. The RNA sequencing was performed at the Genomics Facility at the University of Birmingham. Briefly, the concentrations of the libraries were determined using Qubit and the average library size was determined using TapeStation (Agilent). The libraries were then diluted to 1.6 pM, and 1% of 20 pM PhiX control was added. The libraries were then loaded onto a flow cell and the sequencing was performed on Illumina NEXTseq.

### Quantification and statistical analysis

Data quality control was performed with FastQC v0.11.5 (55), and aligned with STAR v2.5.3a (56) to the human genome (GRCh38.p10, Gencode comprehensive annotation). RNA-seq reads were aligned with parameters; —outSAMtype BAM SortedByCoordinate. The counts per gene were calculated with LiBiNorm v2.4 (57), in HTseq-count (58) compatible mode with parameters; -htseq-compatible, -order pos, -stranded no, -type gene, -idattr gene_name. Count per million normalized bigwig files were constructed using deeptools (59) bamcompare with parameters; -binSize 15, -normalizeUsing CPM. Once raw counts were extracted, the data were analyzed in R (version 4.0.5). Differential expression analysis was performed using DESeq2 package. A gene was considered differentially expressed when the adjusted *P*-value was smaller than 0.1 which is a standard setting for this package. The set of differentially expressed genes was then used as input for the GO analysis, which was executed using top GO package. All of the detected genes were used as input for GSEA analysis which was done using clusterProfiler package.

## Supplementary Material

Supplement_HMG-2022-CE-00220_Grzechnik_ddac228

## Data Availability

The dataset generated during this study is available at GEO (GSE174043).

## Resource availability

Further information and requests for resources and reagents should be directed to and will be fulfilled by the lead contact, Pawel Grzechnik (pawel.grzechnik@manchester.ac.uk).
