# Supplementary material for: Non-coding RNAs associated with Prader–Willi syndrome regulate transcription of neurodevelopmental genes in human induced pluripotent stem cells
Source: Hum Mol Genet. 2022 Sep 9;32(4):608–20. doi: 10.1093/hmg/ddac228 (PMC9896466; doi:10.1093/hmg/ddac228)
Supplement: Supplement_HMG-2022-CE-00220_Grzechnik_ddac228 [file supplement_hmg-2022-ce-00220_grzechnik_ddac228.pdf]

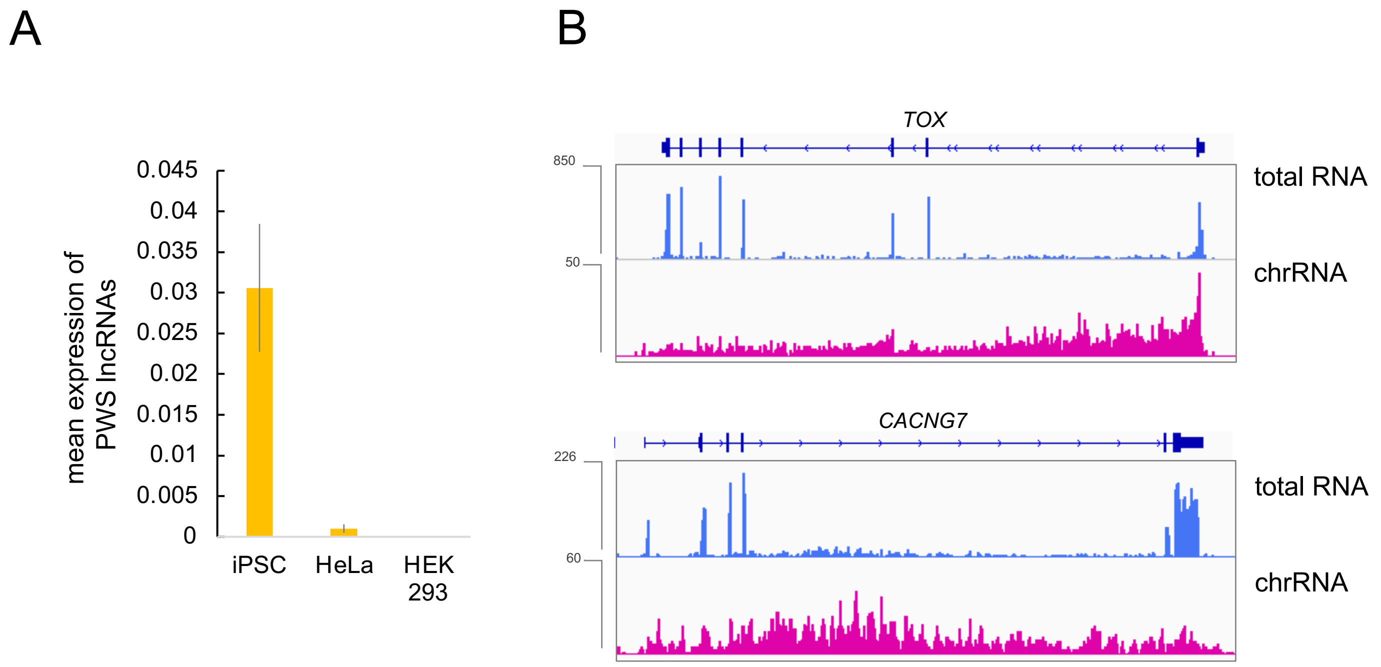

Fig. S1. **(A)** Accumulated expression of PWS lncRNAs in iPSCs, HEK293T and HeLa cells relative to the house-keeping gene *GAPDH*. The average of three biological experiments is shown, error bars indicate standard error. **(B)** Reads coverage for *TOX* and *CACNG7* genes in total and chromatin-associated RNA (chrRNA) samples showing enrichment of reads in the intronic regions in chrRNA, characteristic for samples containing nascent RNA. chrRNA-seq analysis. Note that the axes have different values for total and chrRNA-seq to better visualise the reads distribution. chrRNA-seq track shows counts x10^6.

Fig. S2

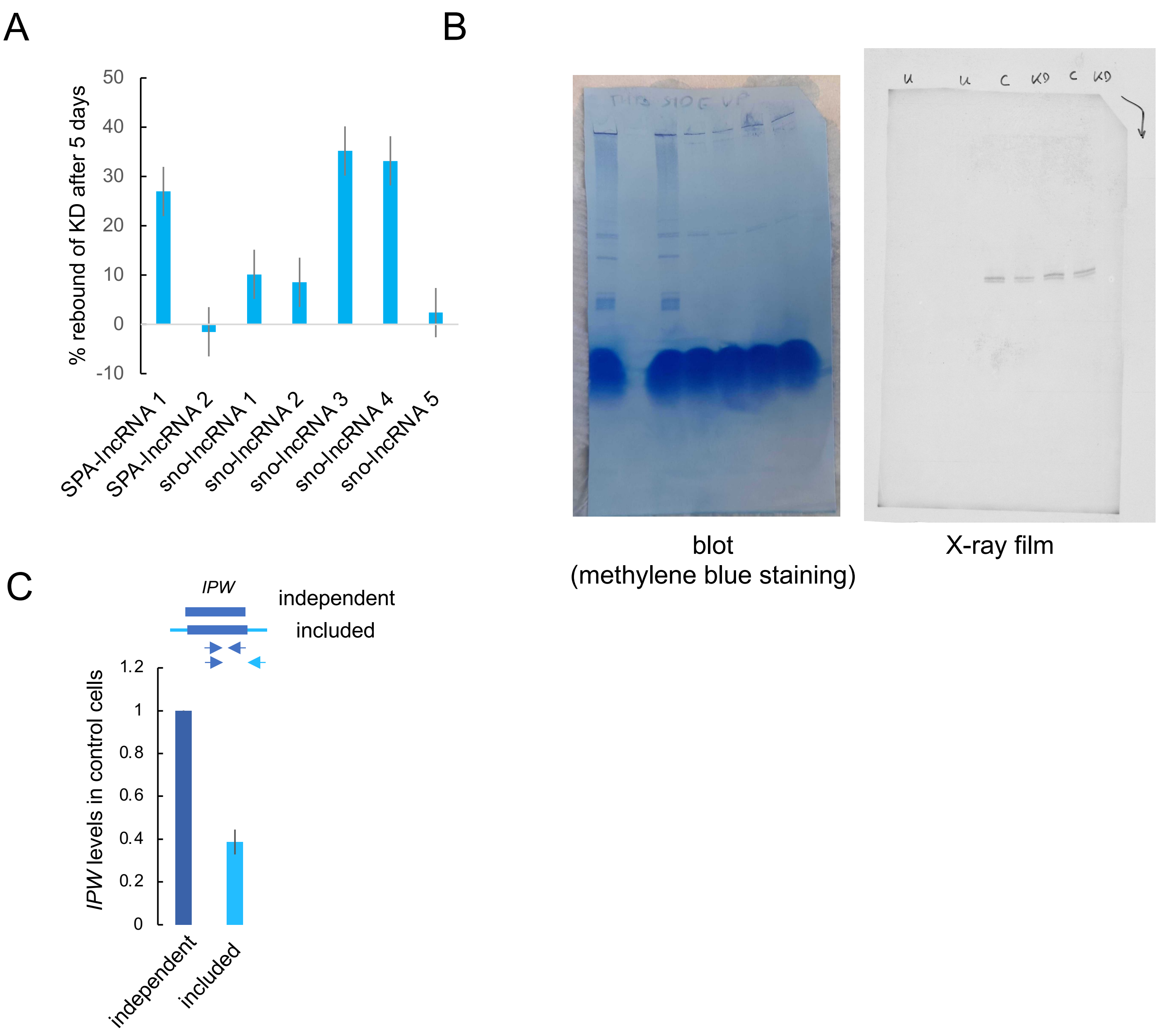

Fig.S2. (A) Percentage increase in expression of sno/SPA-lncRNAs between 24 h and 5 days post knockdown assessed by RT-qPCR analysis. **(B)** Uncropped images of the northern blot and the radiography film. **(C)** The ratio of *SNHG14*-included to independent *IPW* RNAs. The arrows mark the primers used in qPCR analysis. Amplicons were normalised to *ACTB* mRNA and DNA template.

Fig. S3

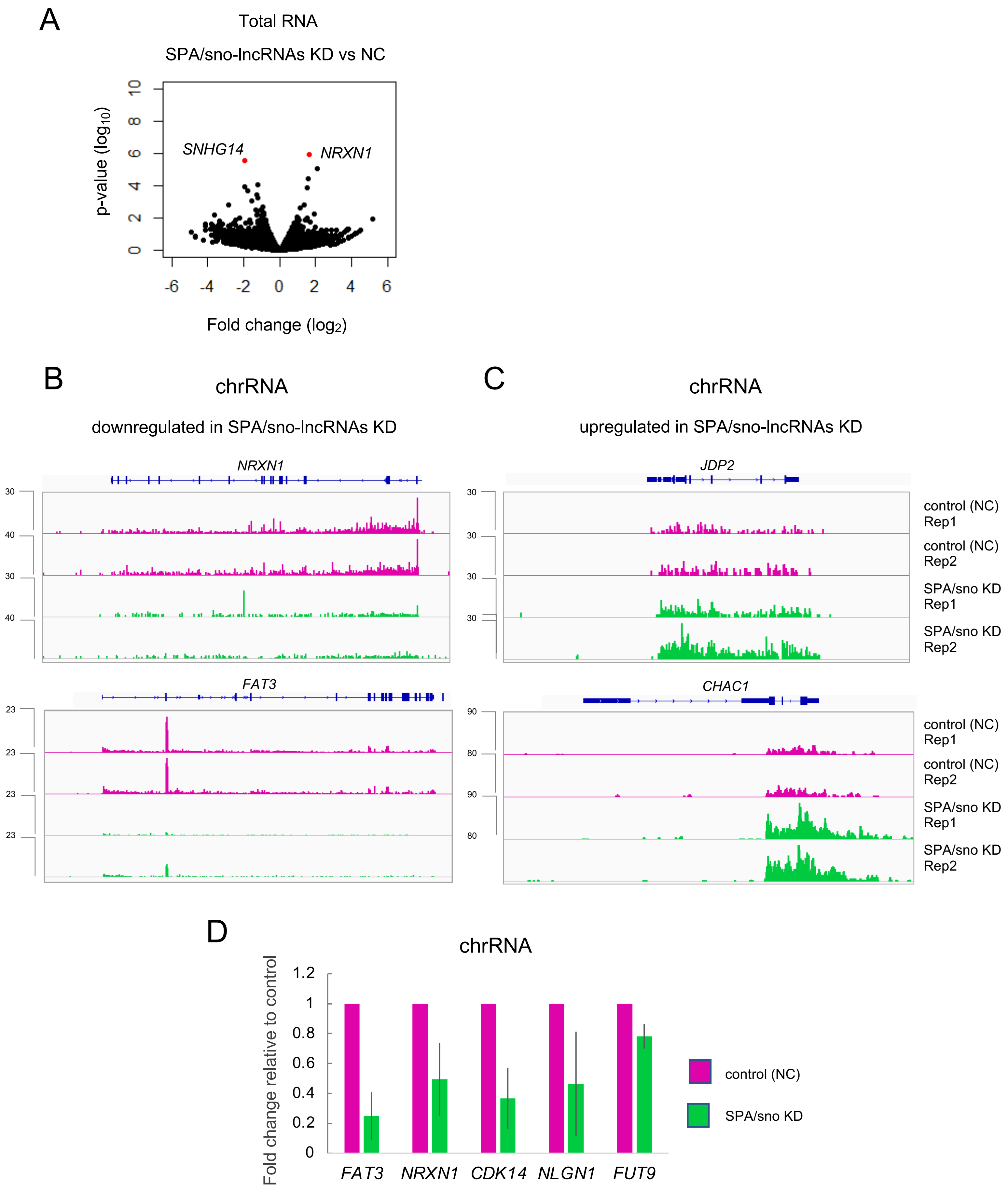

Fig. S3. (A) Volcano plot showing differentially expressed genes (red) between control and SPA/sno-lncRNAs knockdown in total RNA fraction. **(B)** chrRNA-seq reads coverage for the top downregulated genes in SPA/sno-lncRNAs KD and control. **(C)** chrRNA-seq reads coverage for the top upregulated genes in SPA/sno-lncRNAs KD and control. chrRNA-seq track shows counts x10^6. **(D)** Relative expression of top downregulated genes in chRNA-seq analysis normalised to *ACTB* mRNA. qPCR analysis showing an average of four independent experiments, error bars correspond to standard error.

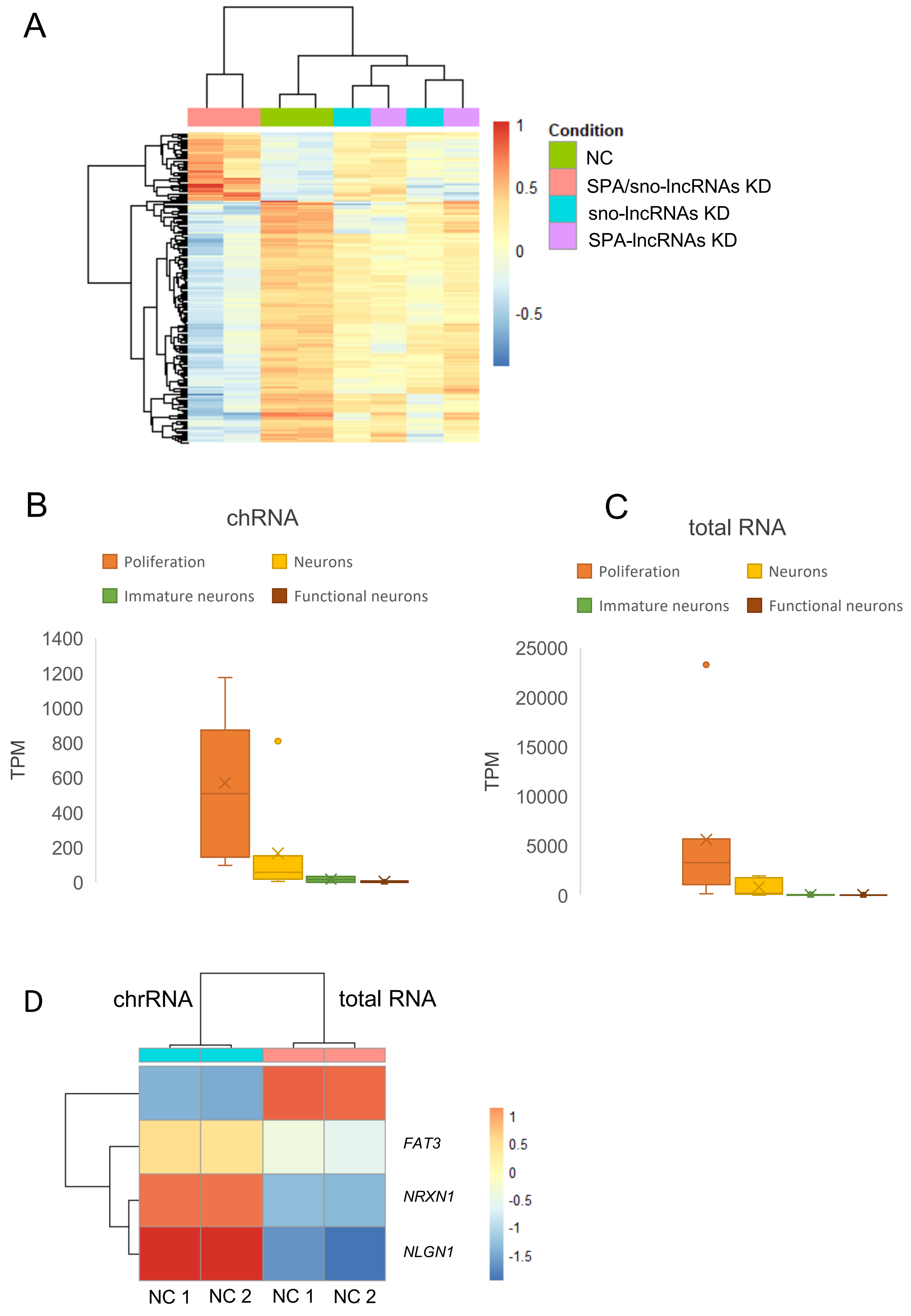

Fig. S4. (A) Heatmap of all of the differentially expressed genes between sno/SPA-lncRNAs KD and control in chrRNA-seq. Note correct clustering between the two replicates of sno/SPA-lncRNAs KD and control samples. **(B-C)** Transcript per million (TPM) values for markers associated with proliferation, immature, mature and functional neurons in control chromatin-associated **(B)** and total **(C)** RNA samples. **(D)** Heatmap showing the expression of neuronal (*FAT3*, *NRXN1*, *NLGN1*) and control (*GAPDH*) genes in total RNA and chromatin-associated RNA fractions in iPSCs.

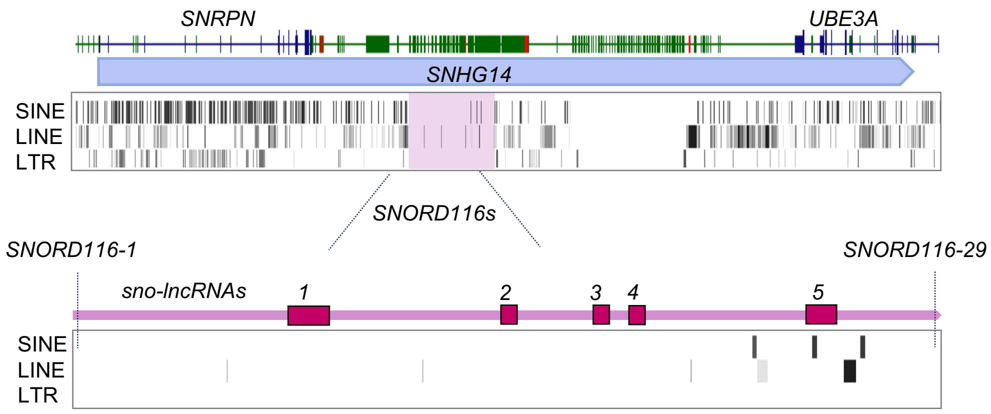

Fig. S5. The distribution of transposon elements in *SNURF-SNRPN/SNHG14* transcription unit.

Table S1. Proliferation, neurons and functional neurons markers.

Proliferation	Neurons	Functional neurons
POU5F1	NEUROD1	CHAT
NANOG	NCAM1	TH
CNMD	DCX	GAD2
USP44	ENO2	SLC17A7
VSNL1	MAP2	SLC17A6
CRABP1	TUBB3	FEV
	NEFL	
	NEFM	
	NEFH	
	GAP43	

Table S2. GapmeRs and oligonucleotides used in the study.

**GapmeRs**	
SPA1	CGATTAGGTTGGAATA
SPA2	CTCGACTGAGAATGAA
sno-lncRNA1	TCAGTAGGATGCGAAT
sno-lncRNA2	AACACCCTTACATCGA
sno-lncRNA3	TCAATGGACGATGAAA
sno-lncRNA4	GCGTGCTTCAGAAAGA
sno-lncRNA5	TGGCTGAATTGGCAAG
PCR primers	
**SPA1**	
SPA1 F	TAATCGGTGGCTTGTCCCTT
SPA1 R	ATGTGCATGGTGGGTGTTTC
**SPA2**	
SPA2 F	AGACTCTCAGAGCCTGGCTC
SPA2 R	CATAAGACAGGATGCTAATG
**sno-lncRNA1**	
sno-lncRNA1 F	CTTGGCGTATTCATGGAGGT
sno-lncRNA1 R	ACCGGCTAAGTGAGCTGAAA
**sno-lncRNA2**	
sno-lncRNA2 F	TGTCCTTGACTCCTGGCTCT
sno-lncRNA2 R	ATGCCAGGTGATTGGAACTC
**sno-lncRNA3**	
sno-lncRNA3 F	CGTGCATCCCTATGTACGTG
sno-lncRNA3 R	CAATGCTACCTGGGAGGTGT
**sno-lncRNA4**	
sno-lncRNA4 F	GGCCAGAGACAGGCAGATAG
sno-lncRNA4 R	GTATCTCCGCAGCTCACACA
**sno-lncRNA5**	
sno-lncRNA5 F	GGAACCAGGGCATAGTGAGA
sno-lncRNA5 R	TTGGATTTGGATGTTGACCA
**SNORD107**	
SNORD107F	GGTTCATGATGACACAGGAC
SNORD107R	GATTTCAGAGTGTCATTTTTAAGC
**SNORD64**	
SNORD64F	GGATTTGTGATGAGCTGTGTT
SNORD64R	GGACTTCAGAGTAATCACG
**SNORD108**	
SNORD108F	GCTTAATGATGAGAATCATTAT
SNORD108R	GACCTCACGCTCCCTTTGCAG
**SNORD109A**	
SNORD109F	GGATCGATGATGAGAATAATT
SNOD109R	GGACCTCAGATTGACATCTG
**SNORD116-1-3**	

SNORD116-1F	GGATCGATGATGAGTCCC
SNORD116-1R	GGACCTCAGTTCCGATGAG
**SNORD116-4**	
SNORD116-1F	GGATCGATGATGAGTCCCCCCA
SNORD116-4R	TGGACCTCAGTTCCGCTGAG
**SNORD116-28**	
SNORD116-28F	TGGATGGATGACGACTTAAAAATG
SNORD116-28R	TTGACCTCAGTTCACAGAACTGC
**IPW**	
IPW 3end F	CTCCCTTAATCAGGACCTTGGTAT
IPW 3end R	TTACACAGGTTTAAGAATTTATTAAG
**IPW-SNHG14**	
IPW 3end F	CTCCCTTAATCAGGACCTTGGTAT
IPW DOG R	CAGGGTAAATCTTTCATTGCCAAG
**SNORD115**	
SNORD115-1F	GTGTTGATGATGAGAACCTTA
SNORD115-1R	GGGCCTCAGCGTAATCCTATT
**FAT3**	
Fat3F	CCTGCGGATTGGGATCTCG
Fat3R	ATCTGCGTCTGTTGCAGTCA
**NRXN1**	
NRXN1 F	TGTTTTGCCGGTGCTGTTAC
NRXN1 R	TGACGCAGATTCCTGGTGAC
**CDK14**	
CDK14 F	AGTTTGGGGAAGTTGTCGGG
CDK14 R	CACAAGTGTCAGCGTCTCCT
**NLGN1**	
NLGN1 F	CCCCGCCTATGATTTTCGGA
NLGN1 R	GCGAAGAGCAAAACTTGGCA
**FUT9**	
FUT9 F	CAGCTCCAGATTCACTGCTCT
FUT9 R	TGCCACTCTTTTGGGGAGTG
